# Different responses to oxidized low-density lipoproteins in human polarized macrophages

**DOI:** 10.1186/1476-511X-10-1

**Published:** 2011-01-04

**Authors:** Kuniaki Hirose, Kazuhisa Iwabuchi, Kazunori Shimada, Takashi Kiyanagi, Chihiro Iwahara, Hitoshi Nakayama, Hiroyuki Daida

**Affiliations:** 1 Department of Cardiovascular Medicine, Juntendo University School of Medicine, 2-1-1, Hongo, Bunkyo-ku, Tokyo 113-8421, Japan; 2 Institute for Environmental and Gender Specific Medicine, Juntendo University Graduate School of Medicine, 2-1-1, Tomioka, Urayasu City, Chiba 278-0021, Japan

## Abstract

**Background:**

Oxidized low-density lipoprotein (oxLDL) uptake by macrophages plays an important role in foam cell formation. It has been suggested the presence of heterogeneous subsets of macrophage, such as M1 and M2, in human atherosclerotic lesions. To evaluate which types of macrophages contribute to atherogenesis, we performed cDNA microarray analysis to determine oxLDL-induced transcriptional alterations of each subset of macrophages.

**Results:**

Human monocyte-derived macrophages were polarized toward the M1 or M2 subset, followed by treatment with oxLDL. Then gene expression levels during oxLDL treatment in each subset of macrophages were evaluated by cDNA microarray analysis and quantitative real-time RT-PCR. In terms of high-ranking upregulated genes and functional ontologies, the alterations during oxLDL treatment in M2 macrophages were similar to those in nonpolarized macrophages (M0). Molecular network analysis showed that most of the molecules in the oxLDL-induced highest scoring molecular network of M1 macrophages were directly or indirectly related to transforming growth factor (TGF)-β1. Hierarchical cluster analysis revealed commonly upregulated genes in all subset of macrophages, some of which contained antioxidant response elements (ARE) in their promoter regions. A cluster of genes that were specifically upregulated in M1 macrophages included those encoding molecules related to nuclear factor of kappa light polypeptide gene enhancer in B-cells (NF-κB) signaling pathway. Quantitative real-time RT-PCR showed that the gene expression of interleukin (IL)-8 after oxLDL treatment in M2 macrophages was markedly lower than those in M0 and M1 cells. *HMOX1 *gene expression levels were almost the same in all 3 subsets of macrophages even after oxLDL treatment.

**Conclusions:**

The present study demonstrated transcriptional alterations in polarized macrophages during oxLDL treatment. The data suggested that oxLDL uptake may affect TGF-β1- and NF-κB-mediated functions of M1 macrophages, but not those of M0 or M2 macrophages. It is likely that M1 macrophages characteristically respond to oxLDL.

## Background

Atherosclerosis is a major cause of cardiovascular disease, which is one of the leading morbidities worldwide [1]. Atherosclerosis has been suggested to be merely a lipid-storage disease; however, it is now recognized as an inflammatory condition of the vessel wall characterized by infiltration of macrophages and T cells [2]. Monocytes are recruited into the arterial intima and differentiate into macrophages. They take up oxidized low-density lipoprotein (oxLDL) *via *scavenger receptors, and then become foam cells that play a crucial role in the initiation of atherosclerotic lesions [3]. Foam cells have been shown to affect many atherogenic events, including recruitment of monocytes and neutrophils by producing chemokines, such as monocyte chemoattractant protein (MCP)-1 [4] and interleukin (IL)-8 [5], formation of necrotic cores in atherosclerotic plaques [3], and production of matrix metalloproteases (MMPs), which degrade the extracellular matrix comprising the fibrous cap of plaque [6]. Therefore, macrophages immunologically interact with surrounding inflammatory cells during the process of differentiation into foam cells in atherogenic processes.

Over the past several decades, a number of studies have demonstrated that macrophages do not represent a homogenous cell population. Stein *et al*. described an alternative subset of macrophages induced by IL-4, characterized by high mannose receptor (MR) expression [7]. Since then, it has been demonstrated that monocyte-derived macrophages can be polarized into two subsets *in vitro*. One subset consists of classically activated macrophages (M1 macrophages) polarized with lipopolysaccharide (LPS) and interferon (IFN)-γ, which are characterized by CD86 expression and production of proinflammatory cytokines, such as tumor necrosis factor (TNF)-α, IL-1, and IL-6. The other subset consists of alternatively activated macrophages (M2 macrophages) polarized with Th2 cytokines, such as IL-4 and/or IL-13, which are characterized by MR expression [8].

Recently, Bouhlel *et al*. confirmed the presence of M2 macrophages within human atherosclerotic lesions by identifying the expression of M2 markers, including IL-10 and MR in human carotid plaques [9]. They also reported that macrophages expressing M2 markers show a different distribution from foam cells. These results suggested the presence of heterogeneous subsets of macrophages in human atherosclerotic lesions. However, it remains unclear which type of macrophages differentiate into foam cells or how they contribute to atherogenesis.

The present study was performed to elucidate the contributions of M1 and M2 macrophages to atherogenesis during differentiation into foam cells. Martinez *et al*. investigated the polarization of human monocytes toward M1 or M2 macrophages using cDNA microarray analysis, and found distinct sets of genes specifically upregulated in either subset of macrophages [10]. Cho *et al*. also examined the transcriptional differences in human monocyte-derived macrophages during oxLDL uptake by cDNA microarray analysis [11]. However, there have been no previous studies of the whole transcriptional alterations in human M1 or M2 macrophages during oxLDL uptake. To investigate the roles of these macrophage subsets during differentiation into foam cells, we examined the transcriptional alterations of M1 or M2 macrophages during oxLDL treatment.

## Methods

### Materials

Lymphoprep was purchased from AXIS-SHILD (Rodelokka, Oslo, Norway). Dulbecco's Modified Eagle's Medium: Nutrient Mixture F-12 (DMEM/F12) was obtained from Invitrogen (Carlsbad, CA), and RPMI-1640 culture medium (endotoxin-free) was from Sigma-Aldrich (St. Louis, MO). Recombinant human macrophage-colony stimulating factor (M-CSF), IFN-γ, and IL-4 were obtained from R&D Systems (Minneapolis, MN). LPS from *Escherichia coli *(serotype O111:B4) was obtained from List Biological Laboratories Inc. (Campbell, CA). OxLDL (endotoxin level < 0.5 EU/ml), which was prepared with 3.5 μM CuSO_4 _in PBS at 37°C for 24 h, was purchased from Biomedical Technologies (Stoughton, MA). The average level of thiobarbituric acid-reactive substances (TBARS) in this study was 76.23 ± 7.89 nmol malondialdehyde equivalents/mg LDL protein (mean ± SD). Anti-CD14 antibody, anti-CD86 antibody, and anti-MR antibody were obtained from eBioscience (San Diego, CA). All procedures were performed under endotoxin-free conditions.

### Cells

Peripheral blood mononuclear cells (PBMCs) were obtained from healthy volunteers with informed consent from buffy coats by density-gradient centrifugation using Lymphoprep. The purity of monocytes was > 95% as determined by flow cytometric analysis using anti-CD14 antibody (data not shown). The monocytes were suspended in DMEM/F12, and plated onto tissue culture dishes at a density of 1 × 10^6 ^cells/cm^2 ^for 2 h at 37°C. The adherent cells were differentiated into macrophages by incubation with 100 ng/mL M-CSF in RPMI-1640 medium supplemented with 20% fetal calf serum (FCS) for 7 days (these cells were defined as M0 macrophages). Macrophage polarization was performed as described by Martinez *et al*. with slight modifications [10]. To obtain M1 or M2 macrophages, M0 macrophages were further incubated with 10 pg/mL LPS plus 20 ng/mL IFN-γ or 20 ng/mL IL-4 in RPMI-1640 with 5% FCS for 18 h, respectively. After polarization, media were removed, and each subset of macrophages was incubated for a further 6 h in the presence or absence of 100 μg/mL oxLDL. The study was approved by the Ethical Committee of Juntendo University.

### Flow cytometric analysis

The M1 or M2 polarized macrophages were washed with PBS. After washing, cells were stained with PE-Cy5- or FITC-conjugated antibodies or with corresponding isotype controls for 20 min at 4°C. Then, flow cytometry was performed to determine the expression of cell surface antigens using FACSCalibur (BD Biosciences, Franklin Lakes, NJ), as described previously [12]. Data were analyzed using Cell Quest software (BD Biosciences).

### Quantitative real-time RT-PCR

Total RNA was extracted and purified from macrophages using an RNeasy Mini Kit (Qiagen, Valencia, CA). cDNA was synthesized from 50 ng/μL of total RNA using an ExScript RT-PCR Kit (Takara-Bio, Shiga, Japan). Primers were selected using Perfect Real-Time Primer Support System provided by Takara. Real-time RT-PCR was performed using SYBR Premix Ex Taq (Takara-Bio) and an ABI 7900HT Sequence Detector System (Applied Biosystems, Foster City, CA). The amplification program included an initial denaturation step at 95°C for 10 s, 40 cycles of denaturation at 95°C for 10 s, and annealing and extension at 60°C for 30 s. After amplification, dissociation curves were acquired to determine the specificity of PCR products. The relative cDNA concentrations were established using a standard curve plotted with sequential tenfold dilutions of cDNA synthesized from QPCR Human Reference Total RNA (Stratagene, La Jolla, CA). The data were normalized relative to peptidylprolyl isomerase A (PPIA) as an internal control.

### cDNA microarray analysis

cDNA synthesis and aminoallyl labeling of RNA were performed using an amino-allyl RNA amplification kit (Sigma-Aldrich) according to the manufacturer's instructions. The Cy3- or Cy5-labeled aminoallyl RNA was concentrated using Microcon YM-30 (Millipore, Bedford, MA), mixed with hybridization buffer supplied with the kit, and denatured at 95°C for 2 min. The hybridization mixture was applied onto a "3D-Gene" human oligo chip 25 k (Toray Industries, Tokyo, Japan), and incubated according to the manufacturer's instructions. After washing and drying the DNA chip slides, the fluorescent signals were quantified by ScanArray Lite (PerkinElmer Life Sciences, Boston, MA) and analyzed using ScanArray Express software. After subtraction of the mean background level, the fluorescence intensity was normalized relative to the mean sample intensity in each chip. Any given gene was analyzed if its normalized intensity was more than 2^-4^. We defined genes showing a change in expression of > 2-fold during oxLDL treatment as significantly up- or downregulated (log_2 _ratios were greater than +1 or less than -1).

### Ingenuity pathway analysis

Ingenuity Pathway Analysis (IPA) software (version 8.7; Ingenuity Systems, Redwood, CA) was utilized to determine the possible biological pathways and intermolecular networks between candidate genes. A detailed description of IPA software can be found on the Ingenuity Systems website http://www.ingenuity.com/. The significantly up- or downregulated genes were overlaid onto a global molecular network developed from information contained in the Ingenuity Knowledge Base.

Functional gene ontology analysis identified the biological functions that were most significant to molecules in the network. The network molecules associated with biological functions in the Knowledge Base were considered for the analysis. Right-tailed Fisher's exact test was used to calculate the *P*-values determining the probability that each biological function assigned to that network was due to chance alone. IPA generates significant biological networks that are particularly enriched with the genes of interest, called "focus genes." It calculates a network score that takes into account the number of focus genes and the size of the networks, indicating the likelihood of focus genes in a network being found together by chance. The higher the score, the lower is the probability of finding the observed Network Eligible Molecules in a given network by chance. Network analysis produces a graphical representation of the molecular relationships between the identified genes. Molecules are represented as nodes, and the biological relationship between two nodes is represented as a line. All relationships are supported by at least 1 reference from the literature, from a textbook, or from canonical information stored in the Knowledge Base.

### Statistical analysis

The data were expressed as the means ± SD and were analyzed for significant differences by one-way or two-way analysis of variance (ANOVA) and, Bonferroni's *post hoc *test using GraphPad Prism (version 5.00; GraphPad Software, La Jolla, CA).

## Results

### Characteristics of M1 or M2 polarized macrophages

**Figure 1:**
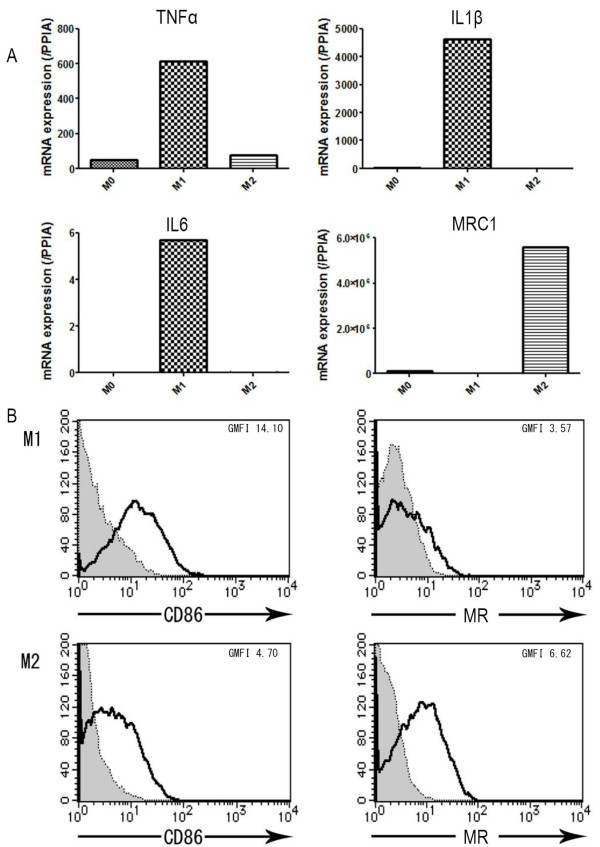
**Expression of M1 and M2 macrophage polarized markers in human monocyte-derived macrophages**. A. *TNFα*, *IL1β*, and *IL6 *gene expression as markers of M1, and *MRC1 *gene expression as a marker of M2 were analyzed by RT-PCR. Each panel shows data from one of 3 representative experiments. B. Surface expression of CD86 (M1 marker) and mannose receptor (MR) (M2 marker) were analyzed by flow cytometry. The level of CD86 expression was higher in M1 than in M2 macrophages, while the expression level of MR was higher in M2 than in M1 macrophages.

Human monocyte-derived macrophages cultured for 7 days in the presence of M-CSF can be polarized toward M1 macrophages by further treatment with 100 ng/mL LPS plus 20 ng/mL IFN-γ for 18 h [10]. However, under our experimental conditions, almost all cells were damaged by such high a concentration of LPS, as demonstrated by trypan blue staining (data not shown). Therefore, we differentiated M-CSF-treated monocytes into M1 macrophages by incubation with 10 pg/mL LPS plus 20 ng/mL IFN-γ. We confirmed the polarized cells as M1 and M2 macrophages by quantitative real-time RT-PCR and flow cytometric analysis (Figure 1). Consistent with the previous report of Martinez *et al*. [10], M1 macrophages showed higher levels of proinflammatory cytokine mRNA expression, such as TNF-α, IL-1β, and IL-6, than M0 or M2 macrophages. In contrast, M2 macrophages showed markedly elevated expression of MRC1, which encodes MR (Figure 1A). The level of CD86 expression on M1 macrophages was higher than that on M2 macrophages, while MR was expressed on M2 macrophages but not on M1 macrophages (Figure 1B).

### Transcriptional profile

**Figure 2:**
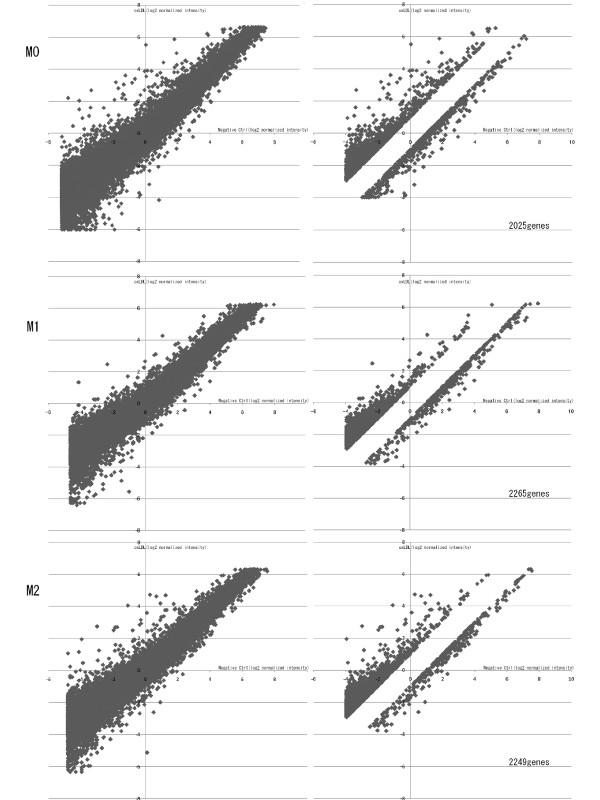
**Genes showing significantly altered expression in oxLDL-treated macrophages on cDNA microarray analysis**. The logarithmically transformed intensities of all the genes in oxLDL-treated macrophages were plotted against those in non-treated macrophages (Left panels). Genes were eliminated if the normalized intensity was less than 2^-4^, or if the change in alteration during oxLDL treatment was less than 2-fold (Right panels). Finally, 2025, 2265, and 2249 genes were identified as showing significantly regulated expression in M0, M1, and M2 macrophages, respectively.

After confirming that the 2 subsets of macrophages were properly polarized to M1 or M2 macrophages, cDNA microarray analysis was performed to investigate the alterations during oxLDL treatment. Of the 25392 probe sets on "3D-Gene" human oligo chip 25 k, we eliminated 1125 probe sets as controls and backgrounds. Any given gene was eliminated if its normalized intensity was less than 2^-4^. We also eliminated non-altered genes that showed changes in expression level of less than 2-fold during oxLDL treatment. Finally, we identified 2025, 2265, and 2249 genes that were significantly up- or downregulated in M0, M1, and M2 macrophages, respectively (Figure 2). Among these genes, 1526, 1819, and 1880 genes were upregulated in M0, M1, and M2 macrophages by oxLDL treatment, respectively (All transcriptional profiles are shown in Additional file 1). Table 1 shows the top 30 genes that were most markedly upregulated by oxLDL. *IL8*, *TRIM16*, and *ADM *were commonly upregulated in all subsets of macrophages. Twenty-eight genes in the top 30 upregulated genes in M2 macrophages (93% of the top 30 genes) were also upregulated in M0, while 15 genes in the top 30 upregulated genes in M2 macrophages were upregulated in M1 cells.

**Table 1:** Genes upregulated by oxLDL in polarized macrophages

The top 30 genes upregulated by oxLDL in M0					
**Gene Symbol**	**Ref Seq ID**	**M0**	**M1**	**M2**	**NCBI official full name**

*IL8*	NM_000584	**6.200**	**3.200**	**5.177**	interleukin 8
*CCL1*	NM_002981	**6.028**	0.937	-	chemokine (C-C motif) ligand 1
*SERPINB2*	NM_002575	**5.485**	-	-	serpin peptidase inhibitor, clade B (ovalbumin), member 2
*IL1B*	NM_000576	**5.162**	**2.700**	**2.684**	interleukin 1, beta
*PTGS2*	NM_000963	**4.985**	-	-	prostaglandin-endoperoxide synthase 2
*TM4SF1*	NM_014220	**4.836**	-	**1.238**	transmembrane 4 L six family member 1
*CCL19*	NM_006274	**4.834**	0.368	-	chemokine (C-C motif) ligand 19
*POPDC3*	NM_022361	**4.557**	**1.491**	-	popeye domain containing 3
*INHBA*	NM_002192	**4.476**	**2.335**	-	inhibin, beta A
*CKB*	NM_001823	**4.378**	0.920	**2.107**	creatine kinase, brain
*IFIT1*	NM_001001887	**4.152**	0.717	**5.734**	interferon-induced protein with tetratricopeptide repeats 1
*CCL5*	NM_002985	**4.075**	-0.218	**1.616**	chemokine (C-C motif) ligand 5
*IFIT5*	NM_012420	**4.039**	-	-	interferon-induced protein with tetratricopeptide repeats 5
*TNFAIP6*	NM_007115	**4.033**	0.019	-	tumor necrosis factor, alpha-induced protein 6
*TRIM16*	NM_006470	**3.990**	**2.992**	**3.453**	tripartite motif-containing 16
*ADM*	NM_001124	**3.973**	**4.807**	**3.202**	adrenomedullin
*CCL4L2*	NM_207007	**3.905**	**1.321**	0.448	chemokine (C-C motif) ligand 4-like 2
*ISG20*	NM_002201	**3.839**	0.952	**4.442**	interferon stimulated exonuclease gene 20kDa
*EREG*	NM_001432	**3.726**	**2.793**	-	epiregulin
*CSF2*	NM_000758	**3.644**	**1.605**	0.724	colony stimulating factor 2 (granulocyte-macrophage)
*RHOF*	NM_019034	**3.638**	-	**3.020**	ras homolog gene family, member F (in filopodia)
*GSTM3*	NM_000849	**3.632**	**2.635**	**2.543**	glutathione S-transferase mu 3 (brain)
*AKR1C3*	NM_003739	**3.497**	**2.490**	**3.432**	aldo-keto reductase family 1, member C3
*CYP7B1*	NM_004820	**3.482**	-	-	cytochrome P450, family 7, subfamily B, polypeptide 1
*CCL4*	NM_002984	**3.466**	0.742	-0.123	chemokine (C-C motif) ligand 4
*CCL24*	NM_002991	**3.433**	0.413	-	chemokine (C-C motif) ligand 24
*NT5E*	NM_002526	**3.406**	-	-	5'-nucleotidase, ecto (CD73)
*IFI44L*	NM_006820	**3.325**	-0.074	**4.474**	interferon-induced protein 44-like
*COX11*	NM_004375	**3.306**	**1.946**	**1.111**	COX11 cytochrome c oxidase assembly homolog (yeast)
*NOL12*	NM_024313	**3.281**	0.937	**1.313**	nucleolar protein 12

**The top 30 genes upregulated by oxLDL in M1**					

**Gene Symbol**	**Ref Seq ID**	**M0**	**M1**	**M2**	**NCBI official full name**

*ADM*	NM_001124	**3.973**	**4.807**	**3.202**	adrenomedullin
*S1RT5*	NM_012241	**3.039**	**3.486**	**2.137**	sirtuin 5
*CXCR5*	NM_032966	**1.986**	**3.437**	**1.815**	chemokine (C-X-C motif) receptor 5
*KLHL21*	NM_014851	**2.380**	**3.324**	-	kelch-like 21 (Drosophila)
*IL8*	NM_000584	**6.200**	**3.200**	**5.177**	interleukin 8
*DPYSL3*	NM_001387	**3.064**	**3.192**	**3.497**	dihydropyrimidinase-like 3
*RAB43*	NM_198490	**1.264**	**3.003**	**3.324**	RAB43, member RAS oncogene familyprovided
*TRIM16*	NM_006470	**3.990**	**2.992**	**3.453**	tripartite motif-containing 16
*C1orf66*	NM_015997	0.382	**2.970**	**1.953**	chromosome 1 open reading frame 66
*PIP4K2A*	NM_005028	**2.325**	**2.967**	**3.127**	phosphatidylinositol-5-phosphate 4-kinase, type II, alpha
*AGAP3*	NM_031946	**1.289**	**2.945**	**1.091**	ArfGAP with GTPase domain, ankyrin repeat and PH domain 3
*INPP4A*	NM_001566	0.129	**2.872**	**1.872**	inositol polyphosphate-4-phosphatase, type I, 107kDa
*DAGLA*	NM_006133	**1.545**	**2.834**	**2.807**	diacylglycerol lipase, alpha
*EREG*	NM_001432	**3.726**	**2.793**	-	epiregulin
*AGAP11*	NM_133447	0.971	**2.791**	**2.318**	ankyrin repeat and GTPase domain Arf GTPase activating protein 11
*ADAMTS10*	NM_030957	0.683	**2.773**	0.952	ADAM metallopeptidase with thrombospondin type 1 motif, 10
*KCP*	NM_199349	**1.269**	**2.746**	**1.861**	kielin/chordin-like protein
*ZFAT*	NM_020863	**1.758**	**2.743**	**2.002**	zinc finger and AT hook domain containing
*LZTR1*	NM_006767	**1.092**	**2.714**	**3.134**	leucine-zipper-like transcription regulator 1
*MON1B*	NM_014940	0.234	**2.712**	-0.754	MON1 homolog B (yeast)
*IL1B*	NM_000576	**5.162**	**2.700**	**2.684**	interleukin 1, beta
*STAM*	NM_003473	-0.222	**2.697**	-0.150	signal transducing adaptor molecule (SH3 domain and ITAM motif) 1
*MAFG*	NM_032711	**2.775**	**2.684**	**2.997**	v-maf musculoaponeurotic fibrosarcoma oncogene homologG (avian)
*OSGIN1*	NM_182981	**2.427**	**2.672**	**2.002**	oxidative stress induced growth inhibitor 1
*ZNF673*	NM_017776	0.606	**2.669**	-	zinc finger family member 673
*IREB2*	NM_004136	-	**2.648**	-	iron-responsive element binding protein 2
*GSTM3*	NM_000849	**3.632**	**2.635**	**2.543**	glutathione S-transferase mu 3 (brain)
*UBR2*	NM_015255	**2.160**	**2.619**	**2.371**	ubiquitin protein ligase E3 component n-recognin 2
*SFT2D3*	NM_032740	**1.146**	**2.577**	**1.609**	SFT2 domain containing 3
*FAM70A*	NM_017938	-	**2.577**	**2.614**	family with sequence similarity 70, member A

**The top 30 genes upregulated by oxLDL in M2**					

**Gene Symbol**	**Ref Seq ID**	**M0**	**M1**	**M2**	**NCBI official full name**

*IFIT2*	NM_001547	**2.559**	-	**5.893**	interferon-induced protein with tetratricopeptide repeats 2
*IFIT1*	NM_001001887	**4.152**	0.717	**5.734**	interferon-induced protein with tetratricopeptide repeats 1
*IL8*	NM_000584	**6.200**	**3.200**	**5.177**	interleukin 8
*MX2*	NM_002463	**2.482**	0.562	**4.552**	myxovirus (influenza virus) resistance 2 (mouse)
*IFI44L*	NM_006820	**3.325**	-0.074	**4.474**	interferon-induced protein 44-like
*ISG20*	NM_002201	**3.839**	0.952	**4.442**	interferon stimulated exonuclease gene 20kDa
*IDO1*	NM_002164	-	0.623	**4.335**	indoleamine 2,3-dioxygenase 1
*PLSCR1*	NM_021105	**2.002**	-0.179	**4.119**	phospholipid scramblase 1
*IFIT3*	NM_001549	**2.306**	-0.483	**3.943**	interferon-induced protein with tetratricopeptide repeats 3
*SGPP2*	NM_152386	**3.229**	-	**3.626**	sphingosine-1-phosphate phosphatase 2
*OR9I1*	NM_001005211	**2.923**	**1.683**	**3.574**	olfactory receptor, family 9, subfamily I, member 1
*VNN1*	NM_004666	0.568	0.896	**3.569**	vanin 1
*DPYSL3*	NM_001387	**3.064**	**3.192**	**3.497**	dihydropyrimidinase-like 3
*TRIM16*	NM_006470	**3.990**	**2.992**	**3.453**	tripartite motif-containing 16
*EPSTI1*	NM_033255	**2.678**	-0.642	**3.439**	epithelial stromal interaction 1 (breast)
*AKR1C3*	NM_003739	**3.497**	**2.490**	**3.432**	aldo-keto reductase family 1, member C3 (3-alpha hydroxysteroid dehydrogenase, type II)
*RAB43*	NM_198490	**1.264**	**3.003**	**3.324**	RAB43, member RAS oncogene family
*APOL6*	NM_030641	**1.925**	0.723	**3.291**	apolipoprotein L, 6
*USP18*	NM_017414	**1.686**	0.052	**3.288**	ubiquitin specific peptidase 18
*ADM*	NM_001124	**3.973**	**4.807**	**3.202**	adrenomedullin
*RSAD2*	NM_080657	**1.858**	0.083	**3.194**	radical S-adenosyl methionine domain containing 2
*LZTR1*	NM_006767	**1.092**	**2.714**	**3.134**	leucine-zipper-like transcription regulator 1
*PIP4K2A*	NM_005028	**2.325**	**2.967**	**3.127**	phosphatidylinositol-5-phosphate 4-kinase, type II, alpha
*CSF2RA*	NM_172247	**1.683**	**2.109**	**3.115**	colony stimulating factor 2 receptor, alpha, low-affinity (granulocyte-macrophage)
*TNFRSF9*	NM_001561	**2.899**	**1.702**	**3.100**	tumor necrosis factor receptor superfamily, member 9
*ARHGAP44*	NM_014859	**1.136**	**1.919**	**3.053**	Rho GTPase activating protein 44
*RHOF*	NM_019034	**3.638**	-	**3.020**	ras homolog gene family, member F (in filopodia)
*CWF19L1*	NM_018294	**1.271**	**2.098**	**3.005**	CWF19-like 1, cell cycle control (S. pombe)
*MAFG*	NM_032711	**2.775**	**2.684**	**2.997**	v-maf musculoaponeurotic fibrosarcoma oncogene homolog G (avian)
*TNFRSF4*	NM_003327	**1.815**	**1.376**	**2.974**	tumor necrosis factor receptor superfamily, member 4

Non-polarized (M0) and polarized M1 and M2 macrophages were treated with or without oxLDL for 6 h. The changes in gene expression were analyzed by cDNA microarray analysis as described in the Materials and methods section. The top 30 upregulated genes in each subset of macrophages are listed. The values denote fold changes (log_2 _ratio) of normalized intensities during oxLDL treatment. Letters in boldface indicate genes that were also upregulated by more than 2-fold in the two other subsets. "-" indicates genes that did not show significantly altered expression. Twenty-eight of the top 30 upregulated genes in M2 macrophages (93% of the top 30 genes) were also upregulated in M0, while 15 genes in the top 30 upregulated genes in M2 macrophages were upregulated in M1 cells.

### Functional gene ontology

**Figure 3:**
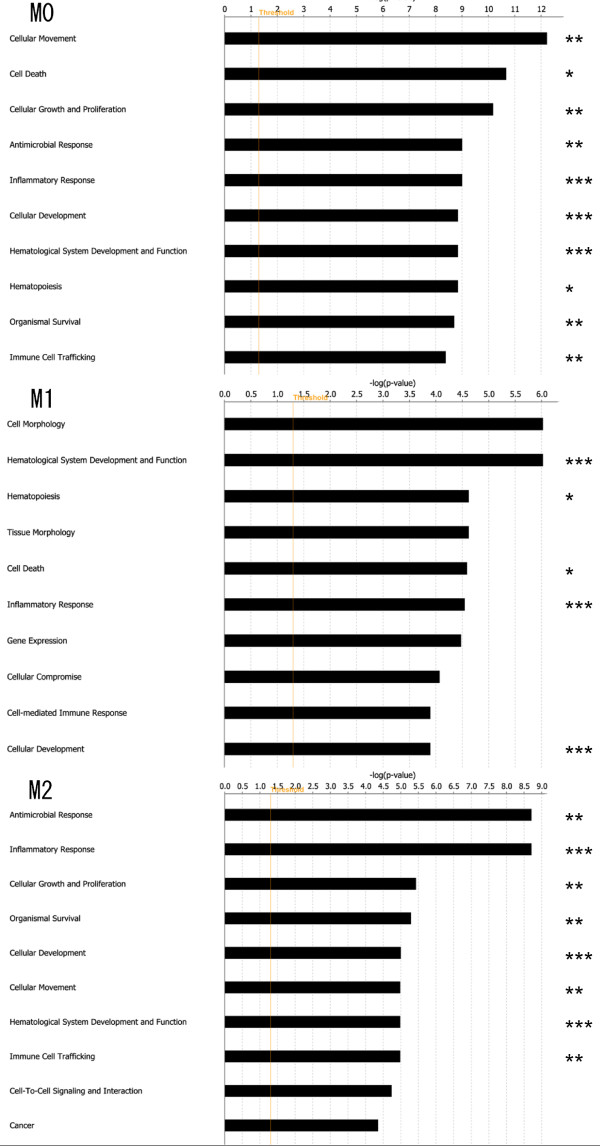
**Gene ontology analysis**. The genes showing significantly upregulated expression in oxLDL-treated macrophages were functionally categorized into groups according to gene ontology. The top 10 functional ontology categories in each subset of macrophages (M0, M1, and M2) are shown in order of *P*-value. Right-tailed Fisher's exact test was used to calculate the *P*-value determining the probability that each biological function assigned to that network was due to chance alone. Eight of the top 10 ontology categories were found in both M0 and M2. *Common in M0 and M1. **Common in M0 and M2. *** Common in M0, M1, and M2.

To identify oxLDL treatment-related biological functions of polarized macrophages, bioinformatics aspects of differentially expressed genes during oxLDL treatment were further analyzed using IPA software. The 1566, 1738, and 1749 genes of M0, M1, and M2 macrophages were identified by IPA software as functionally intentional genes, and categorized into 65, 84, and 80 groups according to functional gene ontology, respectively. Figure 3 shows the top 10 functional ontology categories which contain the molecules altered by oxLDL treatment. Eight of the top 10 ontology categories of M0 macrophages were also found in the top 10 of M2 macrophages, whereas only 3 ontology categories of M1 macrophages were found in the top 10 of M2.

### Molecular network analysis

**Figure 4:**
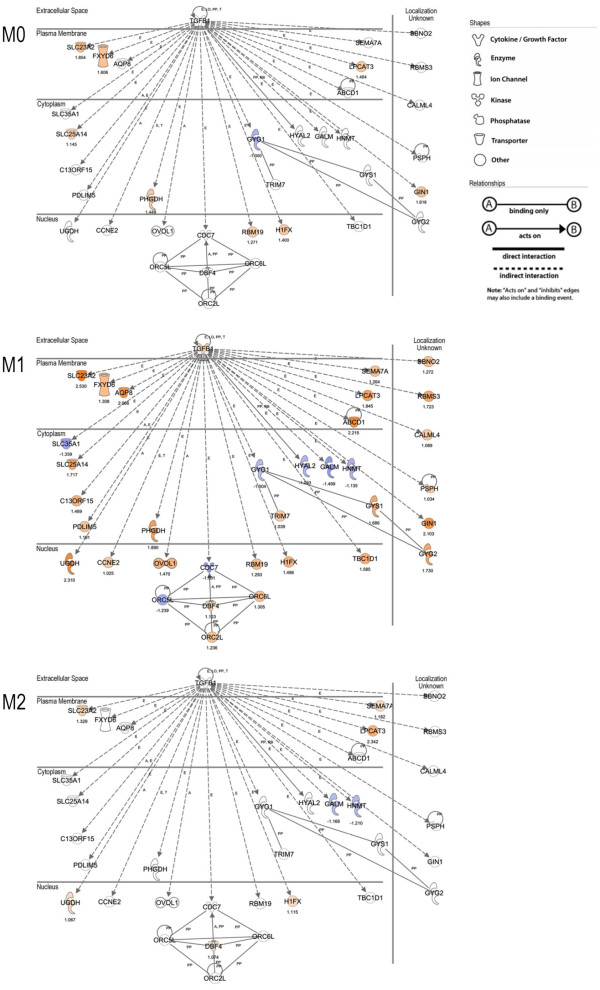
**Molecular network analysis of the highest scoring network in Table 2**. The molecular network in network #1 of M1 macrophages shown in Table 2 and corresponding data of M0 and M2 macrophages are shown. Nearly all the molecules in M1 were related to TGF-β1 directly or indirectly. Molecules are represented as nodes, and the biological relationships between pairs of nodes are represented as lines. The intensity of the node color indicates the degree of upregulation (red) or downregulation (blue). The numbers below the nodes denote fold changes (log_2 _ratio) of normalized intensities during oxLDL treatment. Nodes are displayed using various shapes representing the functional class of the gene product. Lines are displayed with various labels describing the nature of the relationship between the nodes; *i.e*., A for Activation, E for Expression, LO for Localization, PD for Protein - DNA binding, PP for Protein - Protein binding, RB for Regulation of Binding, and T for Transcription.

We performed molecular network analysis using IPA software to elucidate the molecular relationships when each subset of macrophages was treated with oxLDL. The top 5 highly scoring networks of each subset are shown in Table 2. Among these networks, the highest scoring network was found in M1 macrophages as Network #1, including molecules related to "carbohydrate metabolism," "DNA replication, recombination and repair," and "embryonic development." Interestingly, most of the molecules in network #1 were related to transforming growth factor (TGF)-β1 directly or indirectly (Figure 4).

**Table 2:** Molecular network analysis using IPA

Top 5 Networks in M0				
**ID**	**Molecules in Network**	**Score**	**Focus Molecules**	**Top Functions**

#1	*BARX2, CD226, CRABP1, CTH, Cytokeratin, FOLR2, FOS, GCLM, GCNT1, HHEX, HTATIP2, IGDCC3, KCNJ2, KRT8, KRT18, KRT20, KRT6A, LPCAT3, MAF, MAFB, MAFF, MAFG, MT1A, musculoaponeurotic fibrosarcoma oncogene, NFE2L3, NRF1, PAX6, PLAGL1, PVR, S100P, TGFBI, TH1 Cytokine, TLE1, TMSB4, TMSB4X*	33	31	Cellular Assembly and Organization, Cellular Function and Maintenance, Hair and Skin Development and Function

#2	*Alpha tubulin, APEX2, ARRDC3, CALB2, Calbindin, CSF1, CXORF21, DHCR24, DUB, EHMT2, FAM105A, FAM107A, GSC, HDAC6, HOOK2, LMO2, LRRC58, MIR124, NTRK3, oxidoreductase, POU5F1, PPP1R12C, REST, RYK, STAU1, TRPM2, TSPAN14, Tubulin, USP12, USP28, USP34, USP41, USP48, USP49, ZBED3*	28	29	Auditory and Vestibular System Development and Function, Genetic Disorder, Metabolic Disease

#3	*ANAPC1, ANAPC2, ANAPC5, APC, ASNS, BIRC3, CD3EAP, CDC20, CHD2, CKAP2, Cpt, CPT2, Cyclin A, DTYMK, E3 RING, EIF5, GLCE, GPC1, HEXIM1, HGF, IMPDH2, Integrin alpha V beta 3, ISG20, JMJD1C, MYL12A, NAMPT, POLR1A, SLC40A1, SPATC1, SPDEF, TMEM158, TOPBP1, UBR2, Vegf, VHL*	28	29	Protein Degradation, Protein Synthesis, Cardiovascular System Development and Function

#4	*ALDH2, ASXL1, C14ORF1, CBX4, CCDC106, CDK5RAP2, Cyclooxygenase, DIO3, DUSP8, FHL1, JDP2, LSM2, MIP1, MYBPC3, P38 MAPK, PCGF2, PHYHIP, PLEKHN1, PORCN, SEMA7A, SENP2, Stat1 dimer, STK36, SUFU, Tnf receptor, TNFRSF9, TNFRSF11A, TNFSF9, TRAF, TRAF1, TRAF5, TRAF2-TRAF5, TRAIP, TREM1, WNT4*	27	28	Cellular Assembly and Organization, Cellular Function and Maintenance, Skeletal and Muscular System Development and Function

#5	*ANKRD29, APEX1, BAG5, CKB, CKMT1B, CNOT3, CNOT6, CNOT8, Creatine Kinase, DNAJA4, DNAJB6, DNAJC15, DNMBP, Fibrinogen, Hsp70, Hsp90, Hsp22/Hsp40/Hsp90, HSPA2, HSPA1A, ICAM1, IFT52, IP6K2, MIR1, Nos, Pka catalytic subunit, PLSCR1, SH3KBP1, SLC25A30, SPRY2, SRXN1, TIMP3, TNKS1BP1, TNPO2, TRIM2, XPNPEP3*	26	27	Cellular Assembly and Organization, Cellular Development, Cellular Growth and Proliferation

**Top 5 Networks in M1**				

**ID**	**Molecules in Network**	**Score**	**Focus Molecules**	**Top Functions**

**#1**	***ABCD1, AQP8, C13ORF15, CALML4, CCNE2, CDC7, DBF4, FXYD6, GALM, GIN1, GYG1, GYG2, GYS1, H1FX, HNMT, HYAL2, LPCAT3, ORC2L, ORC5L, ORC6L, OVOL1, PDLIM5, PHGDH, PSPH, RBM19, RBMS3, SBNO2, SEMA7A, SLC23A2, SLC25A14, SLC35A1, TBC1D1, TGFB1, TRIM7, UGDH***	**41**	**35**	**Carbohydrate Metabolism**, **DNA Replication, Recombination, and Repair**, **Embryonic Development**

#2	*ATAD3B, CAPZB, CHTF18, CPD, CPT2, FXC1, FXYD1, GAMT, GPC1, GTF3C2, GTF3C4, HMG CoA synthase, HMGCS1, HNRNPA0, IQGAP3, IREB2, LMNB2, LSM2, LSM6, LSM7, MINA, Mir125b (mouse), MYC, Ndpk, NME2, NME6, NME7, PDS5B, RAD21, RANBP6, REC8, RFX2, RNGTT, RRM2B, TOR2A*	34	32	Cellular Assembly and Organization, Genetic Disorder, Metabolic Disease

#3	*ACTN4, APEX2, ATRX, DNA-directed DNA polymerase, Erm, HOOK2, ICAM1, IGSF8, LAS1L, LIG3, LIG4, LRSAM1, MARCH3, MYOZ1, NEIL1, NOM1, PNKP, POLE2, POLG2, POLM, RNF2, RNF6, RNF10, RNF25, RNF166, RNF185, SLC9A1, TRIM37, TRP, TRPC4, UBE2D2, UBE2E3, UBE2 H (includes EG:7328), VIL1, ZNRF1*	34	32	DNA Replication, Recombination, and Repair, Gene Expression, Cell-mediated Immune Response

#4	*ARL15, BRD1, BTBD2, C14ORF1, C1ORF103, CCDC106, CCDC90B, CD3EAP, CDKN3, DNA-directed RNA polymerase, EGLN1, EHMT2, FEZ1, FXR1, GTF3A, histone-lysine N-methyltransferase, KBTBD7, KIF11, KIF2C, KLK10, LMO2, MELK, POLR1A, POLR3 D, POLR3E, POLR3F, RIT1, SETDB1, S*TAB2, *THAP8 (includes EG:199745), TLE1, TMSB4X, UNC119, Vegf, ZNF24 (includes EG:7572)*	30	32	Cell Cycle, Cellular Assembly and Organization, DNA Replication, Recombination, and Repair

#5	*ABCC1, ABCD3, ACIN1, ATP5B, ATPase, CRISPLD2 (includes EG:83716), DTX1, DUSP1, E2F1, FABP1, FAM177A1, GRAP, IL34, Immunoglobulin, INHBA, INHBC, LIF, MAFF, MARK4, MIR124, MYH9, NAA15, NOTCH1, POMC, RDH10, RSF1, RYK, SKIV2L, SLC22A18AS, S*TAB1, *SYNGR2, Trypsin, WRN (includes EG:7486), WWP1, ZNF790*	30	31	Cellular Development, Nervous System Development and Function, Hematological System Development and Function

**Top 5 Networks in M2**				

**ID**	**Molecules in Network**	**Score**	**Focus Molecules**	**Top Functions**

#1	*AKR1C3, ARL15, C1ORF103, CETN3, DCP1A, DCTPP1, DDX6, DDX20, DNA-directed RNA polymerase, DUB, ETV3, FILIP1L, FXYD6, GANAB, GSTM3 (includes EG:2947), GTF3C4, GXYLT1, JMJD1C, NUP155, POLR1A, POLR1E, POLR3F, PRMT1, RHOF, RIF1, SDCCAG8, SNRPA1, TARS, TRAF6, UNC119, USP20, USP41, USP45, VHL, ZFP161*	36	33	Infection Mechanism, Infectious Disease, Cell Cycle

#2	*ATN1, BANP, C8ORF4, CBFA2T2, CLK2, CLOCK, CRY1, DBP, EIF2B1, Fascin, H1FX, HIST1H2AE (includes EG:3012), Histone H1, HNRNPL, Importin beta, KHDRBS3, KIAA0913, LBR, LTBP4, LTC4 S, MAST2, OGG1, Pkc(s), RNF19A, SAFB, SDCBP2, SLC20A2, SRPK1, STK36, SUFU, TIMELESS, TM4SF1, TPM2, XPC, ZNF652*	32	31	Behavior, Nervous System Development and Function, Lipid Metabolism

#3	*ATF7IP, ATPase, BACE1, CAPN3, CASP2, CASP7, CASP10, Caspase, CTRL, ENTPD2, hydrolase, ICAM1, IDE, ITM2C, KIAA1632, KYNU, LGMN, MAGED1, MEOX2, NAIP, NANP, NGFR, NUP160, PAPPA, peptidase, PLEKHF2 (includes EG:79666), RECQL5, RTN3, SENP5 (includes EG:205564), SPPL2B, SPTBN1, THTPA, UBAC2, UBR5, XAF1*	32	31	Protein Degradation, Protein Synthesis, Post-Translational Modification

#4	*API5, ARRDC3, ASPH, Calbindin, CKB, CNNM4, COX11, COX15, COX10 (includes EG:1352), CPT1B, Creatine Kinase, Cytochrome c oxidase, ENO3, FICD, FOXP1, GPRASP2, HTR2B, HTT, JAKMIP1, KLF16, NDUFS1, NEFH, PHB (includes EG:5245), PPP1R16B, RCOR2, REST, RGS14, RNF34, SASH1, SCN4B, SNN, STRN, Thymidine Kinase, VAPA, WAC*	32	31	Gene Expression, Neurological Disease, Cellular Compromise

#5	*ALDH7A1, BPI, Cbp, Ciap, CLIP2, CST7, DBT, DUSP2, G0S2, GFPT2, KLF3, KRT19, lymphotoxin-alpha1-beta2, MICA, NFkB (complex), NKIRAS1, PELI3, POU2F2, RBCK1, REL/RELA/RELB, RNF216, S100P, S100Z, SH3RF1, SIAH2, SLC2A6, SNIP1, SUMO4, TNIP2, TRIM32, TRIM69, TXNRD1, WTAP, ZMYND11, ZNF71*	30	30	Carbohydrate Metabolism, Lipid Metabolism, Small Molecule Biochemistry

Molecular network analysis was performed using IPA software. The top 5 groups of molecules in the network are listed in order of network score, which IPA calculates as shown below. We focused on the highest scoring network #1 in M1 macrophages, which had a score of 41 and included 35 molecules matched with the Ingenuity Knowledge Base.

Network Score = -log_10_(*P*-value)

### Hierarchical cluster analysis

**Figure 5:**
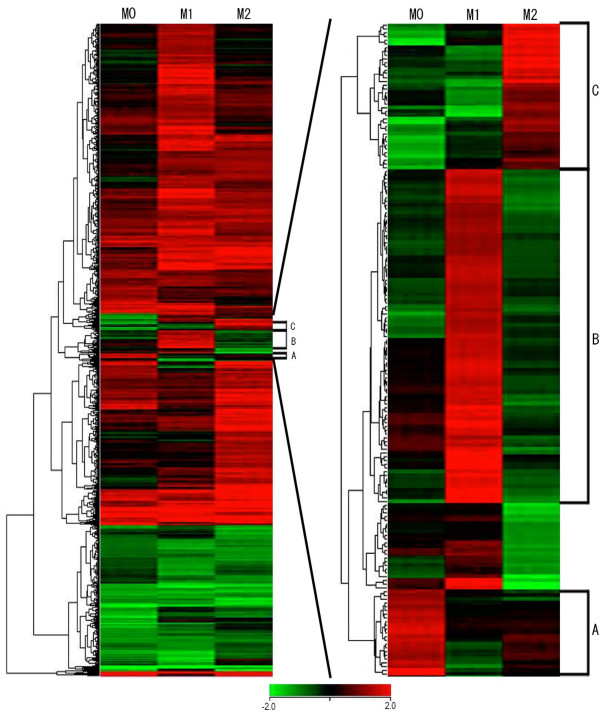
**Heat map constructed by hierarchical cluster analysis**. Red and green in the heat maps indicate up- and downregulation during oxLDL treatment, respectively. The left map includes 3196 genes expression levels of which were significantly altered by oxLDL treatment in at least one subset of macrophages. Genes in three clusters, denoted as A to C, were specifically upregulated only in one subset. There were 17, 72, and 28 annotated genes in cluster A, B, and C, respectively (right map).

Hierarchical cluster analysis allows us to visually comprehend differential patterns over multiple microarray datasets. To analyze the hierarchical clusters over subsets of macrophages, we constructed a heat map where red and green indicated up- and downregulation, respectively (Figure 5). We employed 3196 genes, expression levels of which were significantly altered by oxLDL treatment in at least one subset of macrophages. **A total of **251 genes were **commonly **identified as upregulated genes in all subsets of macrophages, including *TRIM16*, *HMOX1*, *TXNRD1*, *GCLM*, and *DUSP1*, all of which contain an antioxidant response element (ARE) in their promoter regions, which serves as a binding site for nuclear factor erythroid 2-related factor 2 (Nrf2). Hierarchical cluster analysis identified 3 clusters the genes of which were upregulated in one subset but not in the other subsets (Figure 5). Cluster A included 17 annotated genes that were upregulated in M0, but not in the other subsets (Table 3). The genes in cluster A belonged to ontology categories including "cell-mediated immune response," "cellular movement," "hematological system development and function," and "immune cell trafficking." There were 72 annotated genes in cluster B, which were specifically upregulated in M1. These 72 genes were related to "gene expression" and "cellular development." They included *NFKB2*, encoding nuclear factor of kappa light polypeptide gene enhancer in B-cells (NF-κB), and *PIK3R4*, encoding phosphoinositide-3-kinase (PI3K), both of which are molecules related to the NF-κB signaling pathway. In cluster C, 28 annotated genes were identified as specifically upregulated in M2. These genes were associated with "carbohydrate metabolism," "lipid metabolism," and "small molecule biochemistry."

**Table 3:** Genes included in each cluster

**Cluster A**.					
**Gene Symbol**	**Ref Seq ID**	**M0**	**M1**	**M2**	**NCBI official full name**

*CHI3L2*	NM_004000	1.170712	0.008113	-0.453072	chitinase 3-like 2
*PARP8*	NM_024615	1.018767	0.147437	-0.206628	poly (ADP-ribose) polymerase family, member 8
*MAP4K4*	NM_145686	1.115230	-0.302140	-0.763189	mitogen-activated protein kinase kinase kinase kinase 4
*CD44*	NM_001001391	1.312520	-0.201936	-0.048693	CD44 molecule
*CYP27B1*	NM_000785	1.111109	-0.055705	-0.015432	cytochrome P450, family 27, subfamily B, polypeptide 1
*SLC1A2*	NM_004171	1.466166	-0.088519	0.225126	solute carrier family 1 (glial high affinity glutamate transporter), member 2
*CKMT1*	NM_020990	1.436353	0.264923	0.338353	creatine kinase, mitochondrial 1B
*MYOD1*	NM_002478	1.665245	0.211625	0.330158	myogenic differentiation 1
*CD40*	NM_001250	1.319905	-0.606033	0.379319	CD40 molecule, TNF receptor superfamily member 5
*PRKAR2B*	NM_002736	1.349370	-0.472458	0.347752	protein kinase, cAMP-dependent, regulatory, type II, beta
*KIAA0913*	NM_015037	1.244477	-0.318545	0.447287	KIAA0913
*SNX10*	NM_013322	1.138265	-0.351416	0.635676	sorting nexin 10
*HHIP*	NM_022475	1.177555	-0.537186	0.506197	hedgehog interacting protein
*BIRC3*	NM_001165	1.499122	-0.583781	-0.065715	baculoviral IAP repeat-containing 3
*ZBTB7C*	NM_001039360	1.293925	-1.001009	0.404020	zinc finger and BTB domain containing 7C
*PACRGL*	NM_145048	2.026548	-0.460488	0.187722	PARK2 co-regulated-like
*CCL23*	NM_005064	2.471357	-0.192407	-0.036793	chemokine (C-C motif) ligand 23

**Cluster B**.					

**Gene Symbol**	**Ref Seq ID**	**M0**	**M1**	**M2**	**NCBI official full name**

*NDN*	NM_002487	-0.169667	1.457695	-0.617320	necdin homolog (mouse)
*FAM129C*	NM_173544	-0.110208	1.321559	-0.841954	family with sequence similarity 129, member C
*NME6*	NM_005793	-0.315804	1.345342	-0.759802	non-metastatic cells 6, protein expressed in (nucleoside-diphosphate kinase)
*TMEM99*	NM_145274	-0.390527	1.497197	-0.676125	transmembrane protein 99
*ATP2B1*	NM_001682	-0.371182	1.535878	-0.824423	ATPase, Ca++ transporting, plasma membrane 1
*KIF1A*	NM_004321	-0.362399	1.333531	-0.928733	kinesin family member 1A
*TMEM188*	NM_153261	-0.375073	1.157188	-0.955238	transmembrane protein 188
*PLEKHG4*	NM_015432	-0.414580	1.052587	-0.706013	pleckstrin homology domain containing, family G (with RhoGef domain) member 4
*NOXA1*	NM_006647	-0.322207	1.015456	-0.592170	NADPH oxidase activator 1
*CUX1*	NM_001913	-0.236206	1.147118	-0.551592	cut-like homeobox 1
*SBNO2*	NM_014963	-0.294540	1.271977	-0.630553	strawberry notch homolog 2 (Drosophila)
*TIGD5*	NM_032862	-0.376362	1.224225	-0.526717	tigger transposable element derived 5
*CTSL2*	NM_001333	-0.428772	1.153800	-0.359408	cathepsin L2
*ZNF24*	NM_006965	-0.385988	1.069071	-0.360632	zinc finger protein 24
*STK32C*	NM_173575	-0.634840	1.124350	-0.544619	serine/threonine kinase 32C
*NUDT17*	NM_001012758	-0.524910	1.111636	-0.519731	nudix (nucleoside diphosphate linked moiety X)-type motif 17
*CALML4*	NM_033429	-0.433422	1.089113	-0.492229	calmodulin-like 4
*C7orf33*	NM_145304	-0.435642	1.106760	-0.512504	chromosome 7 open reading frame 33
*FBXO22*	NM_012170	-0.203452	1.309369	-0.481216	F-box protein 22
*LINGO1*	NM_032808	-0.174931	1.312372	-0.445152	leucine rich repeat and Ig domain containing 1
*ZMYND19*	NM_138462	-0.243665	1.351306	-0.517691	zinc finger, MYND-type containing 19
*CCR1*	NM_001295	-0.165232	1.419892	-0.480175	chemokine (C-C motif) receptor 1
*ATRX*	NM_000489	-0.498685	1.285463	-0.371568	alpha thalassemia/mental retardation syndrome X-linked
*FRMD4B*	NM_015123	-0.477381	1.252296	-0.287931	FERM domain containing 4B
*CEP164*	NM_014956	-0.419638	1.269277	-0.283263	centrosomal protein 164kDa
*LANCL2*	NM_018697	-0.393507	1.324253	-0.242555	LanC lantibiotic synthetase component C-like 2 (bacterial)
*UBA5*	NM_024818	-0.478021	1.390785	-0.479541	ubiquitin-like modifier activating enzyme 5
*GGCX*	NM_000821	-0.380201	1.330677	-0.476370	gamma-glutamyl carboxylase
*FOXN3*	NM_005197	-0.772837	1.196028	-0.268613	forkhead box N3
*TP53BP1*	NM_005657	-0.797822	1.249895	-0.460222	tumor protein p53 binding protein 1
*ORC6*	NM_014321	-0.633454	1.305326	-0.322922	origin recognition complex, subunit 6
*ST7*	NM_018412	-0.678132	1.309238	-0.290689	suppression of tumorigenicity 7
*OGFOD1*	NM_018233	0.061541	1.413933	-0.438702	2-oxoglutarate and iron-dependent oxygenase domain containing 1
*UBE2B*	NM_003337	0.146284	1.431435	-0.295974	ubiquitin-conjugating enzyme E2B (RAD6 homolog)
*GALNTL4*	NM_198516	0.178950	1.470650	-0.270045	UDP-N-acetyl-alpha-D-galactosamine:polypeptide N-acetylgalactosaminyltransferase-like 4
*HSPA14*	NM_016299	0.185892	1.419392	-0.264205	heat shock 70kDa protein 14
*PEX13*	NM_002618	0.137507	1.527385	-0.274508	peroxisomal biogenesis factor 13
*ATP2B2*	NM_001001331	0.084953	1.689567	-0.394052	ATPase, Ca++ transporting, plasma membrane 2
*TAAR2*	NM_014626	0.168770	1.695874	-0.439255	trace amine associated receptor 2
*HUS1*	NM_004507	0.098273	1.666199	-0.226370	HUS1 checkpoint homolog (S. pombe)
*RIC8B*	NM_018157	0.214093	1.650594	-0.307883	resistance to inhibitors of cholinesterase 8 homolog B (C. elegans)
*DNAJC17*	NM_018163	0.172153	1.482705	-0.492935	DnaJ (Hsp40) homolog, subfamily C, member 17
*TMEM130*	NM_152913	0.206368	1.399311	-0.480833	transmembrane protein 130
*AP2A2*	NM_012305	0.285524	1.391232	-0.458091	adaptor-related protein complex 2, alpha 2 subunit
*TTTY13*	NR_001537	0.381981	1.560081	-0.451429	testis-specific transcript, Y-linked 13 (non-protein coding)
*AKT1S1*	NM_032375	0.385837	1.517956	-0.371995	AKT1 substrate 1 (proline-rich)
*WNT3*	NM_030753	0.009319	1.560600	-0.801987	wingless-type MMTV integration site family, member 3
*ZNF443*	NM_005815	0.154517	1.457293	-0.742146	zinc finger protein 443
*TFCP2L1*	NM_014553	0.207710	1.525949	-0.955561	transcription factor CP2-like 1
*KIAA0355*	NM_014686	0.278629	1.724807	-0.576470	KIAA0355
*ASAP2*	NM_003887	0.524513	1.733462	-0.459752	ArfGAP with SH3 domain, ankyrin repeat and PH domain 2
*C14orf49*	NM_152592	0.578042	1.624871	-0.365254	chromosome 14 open reading frame 49
*NFKB2*	NM_002502	0.652839	1.882528	-0.343188	nuclear factor of kappa light polypeptide gene enhancer in B-cells 2 (p49/p100)
*ZNF586*	NM_017652	0.328381	1.848104	-0.284595	zinc finger protein 586
*RNMTL1*	NM_018146	0.277170	1.645924	-0.133286	RNA methyltransferase like 1
*FOSL2*	NM_005253	0.386974	1.569486	-0.229451	FOS-like antigen 2
*SATB1*	NM_002971	0.542138	1.252296	-0.749110	SATB homeobox 1
*ASB6*	NM_177999	0.666548	1.536652	-0.584820	ankyrin repeat and SOCS box-containing 6
*SLC5A3*	NM_006933	0.682674	1.388148	-0.424733	solute carrier family 5 (sodium/myo-inositol cotransporter), member 3
*C8orf84*	NM_153225	0.637255	1.709652	-0.981619	chromosome 8 open reading frame 84
*MON1B*	NM_014940	0.234404	2.712191	-0.753618	MON1 homolog B (yeast)
*PIGO*	NM_152850	0.134053	2.574539	-0.004491	phosphatidylinositol glycan anchor biosynthesis, class O
*STAM*	NM_003473	-0.221822	2.697280	-0.150394	signal transducing adaptor molecule (SH3 domain and ITAM motif) 1
*CCDC45*	NM_138363	-0.046055	2.118025	-0.312274	coiled-coil domain containing 45
*GYS1*	NM_002103	-0.699205	1.685994	-0.629003	glycogen synthase 1 (muscle)
*RENBP*	NM_002910	-0.403657	1.957579	-0.580833	renin binding protein
*BTD*	NM_000060	-0.348646	1.647239	-0.685758	biotinidase
*FAM172A*	NM_032042	-0.409833	1.717425	-0.665763	family with sequence similarity 172, member A
*PIK3R4*	NM_014602	-0.188899	2.325311	-0.729078	phosphoinositide-3-kinase, regulatory subunit 4
*RSAD1*	NM_018346	-0.251002	2.260460	-0.748812	radical S-adenosyl methionine domain containing 1
*ZNF626*	NM_145297	-0.252970	1.842391	-0.910235	zinc finger protein 626
*CTH*	NM_001902	-1.060839	2.365953	-1.038817	cystathionase (cystathionine gamma-lyase)

**Cluster C**.					

**Gene Symbol**	**Ref Seq ID**	**M0**	**M1**	**M2**	**NCBI official full name**

*MXD3*	NM_031300	-0.800223	0.116059	1.984245	MAX dimerization protein 3
*CD14*	NM_000591	-1.193454	-0.266058	2.053786	CD14 molecule
*BARX1*	NM_021570	-1.650218	0.236670	1.728783	BARX homeobox 1
*ZNF331*	NM_018555	-1.729498	-0.028187	1.786943	zinc finger protein 331
*GPR54*	NM_032551	-0.085064	-0.929503	1.669680	KISS1 receptor
*ANP32A*	NM_006305	-0.155917	-0.929503	1.827632	acidic (leucine-rich) nuclear phosphoprotein 32 family, member A
*C19orf54*	NM_198476	-0.205921	-0.803972	1.634746	chromosome 19 open reading frame 54
*G0S2*	NM_015714	-0.367904	-0.858834	2.131860	G0/G1switch 2
*SPATA3*	NM_139073	-0.301818	-0.432004	1.766064	spermatogenesis associated 3
*FOXA2*	NM_153675	-0.306075	-0.533883	1.983144	forkhead box A2
*PSORS1C2*	NM_014069	-0.423428	-0.713119	1.449567	psoriasis susceptibility 1 candidate 2
*SNN*	NM_003498	-0.492216	-1.239138	1.185086	stannin
*CDKN2B*	NM_078487	-0.404103	-0.847300	1.024029	cyclin-dependent kinase inhibitor 2B (p15, inhibits CDK4)
*ZNF257*	NM_033468	-0.916132	-1.388935	1.189490	zinc finger protein 257
*ZBTB4*	NM_020899	-0.296438	-1.533325	0.937391	zinc finger and BTB domain containing 4
*CASP2*	NM_032983	-1.527530	-0.751505	1.026536	caspase 2, apoptosis-related cysteine peptidase
*TCTEX1D1*	NM_152665	-1.661006	-0.348442	1.280638	Tctex1 domain containing 1
*FILIP1L*	NM_014890	-0.967885	-0.212315	1.018932	filamin A interacting protein 1-like
*USP52*	NM_014871	-0.885665	-0.310932	1.097774	PAN2 poly(A) specific ribonuclease subunit homolog (S. cerevisiae)
*SETD8*	NM_020382	-1.020324	-0.599401	0.697655	SET domain containing (lysine methyltransferase) 8
*C13orf31*	NM_153218	-1.442594	-0.330041	0.688789	chromosome 13 open reading frame 31
*POLD1*	NM_002691	-1.311086	-0.314006	0.812769	polymerase (DNA directed), delta 1, catalytic subunit 125kDa
*DNMT3A*	NM_022552	-1.207327	-0.289399	0.489584	DNA (cytosine-5-)-methyltransferase 3 alpha
*TGFBI*	NM_000358	-1.324217	-0.324120	0.308245	transforming growth factor, beta-induced, 68kDa
*ANP32C*	NM_012403	-1.493138	-0.380761	0.494101	acidic (leucine-rich) nuclear phosphoprotein 32 family, member C
*ITGA2B*	NM_000419	-1.109218	0.018385	1.026536	integrin, alpha 2b (platelet glycoprotein IIb of IIb/IIIa complex, antigen CD41)
*RGS3*	NM_130795	-1.022299	-0.038087	0.656812	regulator of G-protein signaling 3provided
*MARCH1*	NM_017923	-1.374411	0.233896	0.729622	membrane-associated ring finger (C3HC4) 1provided

**The top functional ontology categories**					

**Cluster A**.			***P*-value**		

Cell-mediated Immune Response			9.46E-06-2.65E-02		
Cellular Movement			9.46E-06-3.76E-02		
Hematological System Development and Function			9.46E-06-3.57E-02		
Immune Cell Trafficking			9.46E-06-2.49E-02		
Cardiovascular Disease			2.64E-05-3.76E-02		

**Cluster B**.			***P*-value**		

Cell Cycle			1.31E-04-3.28E-02		
Cell Cycle			1.31E-04-3.28E-02		
DNA Replication, Recombination, and Repair			1.31E-04-4.51E-02		
Gene Expression			2.16E-03-3.35E-02		
Cellular Development			3.51E-03-3.64E-02		
Nervous System Development and Function			3.51E-03-4.7E-02		

**Cluster C**.			***P*-value**		

Carbohydrate Metabolism			1.12E-04-4.09E-02		
Lipid Metabolism			1.12E-04-4.09E-02		
Small Molecule Biochemistry			1.12E-04-4.09E-02		
Cell Death			3.76E-04-4.81E-02		
Nervous System Development and Function			7.79E-04-3.33E-02		

Three clusters were extracted from the heat map shown in Figure 5. There were 17, 72, and 28 annotated genes in clusters A, B, and C, respectively. The top functional ontology categories and the corresponding *P*-values in each subset are shown. *NFKB2 *and *PIK3R4 *were found in cluster B. The values denote fold changes (log_2 _ratio) of normalized intensities during oxLDL treatment.

### Quantitative real-time RT-PCR analysis

All data from cDNA microarray analysis in the present study were comparisons between cells with and without oxLDL treatment. To compare gene expression levels among subsets of macrophages, we performed quantitative real-time RT-PCR for two genes: *IL8 *listed in the top 30 genes that were commonly upregulated in all subsets of macrophages (Table 1) and *HMOX1 *encoding heme oxygenase (HO)-1 as a representative of genes containing an ARE.

**Figure 6:**
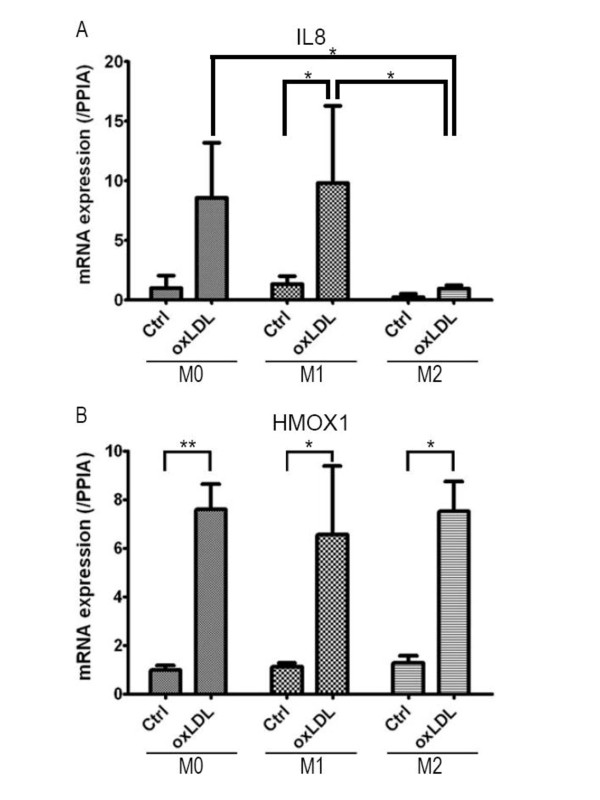
**Quantitative real-time RT-PCR**. A. *IL8 *mRNA expression levels. The expression level of *IL8 *was significantly upregulated by oxLDL treatment in M1 macrophages (*P *< 0.05). *IL8 *expression level after oxLDL treatment in M2 was markedly lower than those in M0 and M1 macrophages (*P *< 0.05). B. *HMOX1 *mRNA expression levels. OxLDL treatment significantly enhanced *HMOX1 *gene expression by 7.6-fold (**P < 0.0005), 5.8-fold (*P < 0.05), and 5.9-fold (*P < 0.05) in M0, M1, and M2 macrophages, respectively. *HMOX1 *gene expression levels were almost the same in all 3 subsets of macrophages even after oxLDL treatment. Each bar shows the mean ± SD of 3 experiments.

Consistent with previous reports, the expression levels of *IL8 *were higher in non-stimulated M1 macrophages than in M2 [10], and oxLDL treatment induced higher levels of *IL8 *expression in M0 macrophages [13,14] (Figure 6A). Moreover, the expression level of *IL8 *was significantly upregulated by oxLDL treatment in M1 macrophages, whereas its expression level after oxLDL treatment in M2 was markedly lower than those in M0 and M1 macrophages (*P *< 0.05). It has been known for several decades that oxLDL treatment increases, while IL-4 treatment decreases IL-8 production in human monocyte-derived macrophages [13,15]. However, a recent report of microarray analysis indicated that oxLDL treatment induced no changes in human monocyte-derived macrophages [11]. This may have been due to various factors, such as individual variations or duration of oxLDL treatment [16].

HO-1 is expressed in vascular endothelial cells and macrophages in the early stages of atherosclerotic lesions and in foam cells in the advanced stages, and is known for its antiinflammatory actions [17,18]. *HMOX1 *is known to be upregulated by oxidized phospholipids in murine macrophages polarized toward M1 or M2 [19]. Treatment with oxLDL yielded markedly higher levels of *HMOX1 *expression in all subsets of macrophages: *i.e*., 7.6-fold (*P *< 0.0005), 5.8-fold (*P *< 0.05), and 5.9-fold (*P *< 0.05) changes in M0, M1, and M2 macrophages compared to corresponding non-treated controls, respectively. *HMOX1 *gene expression levels were almost the same in all 3 subsets of macrophages even after oxLDL treatment (Figure 6B).

## Discussion

In the present study, we demonstrated transcriptional alterations during oxLDL treatment, which has been suggested to be a model of the early stages of foam cell formation, in human polarized macrophages. Our study demonstrated that: 1) 93% of the top 30 genes upregulated by oxLDL treatment in M2 macrophages were also upregulated in M0; 2) the top 10 functional ontology categories in M2 macrophages were similar to those in M0; 3) almost all of the molecules in the highest scoring molecular network of M1 were related either directly or indirectly to TGF-β1; 4) there were commonly upregulated genes in all subset of macrophages, some of which contained ARE in their promoter regions; 5) hierarchical cluster analysis revealed a cluster specifically upregulated in M1, including genes encoding molecules related to the NF-κB signaling pathway; 6) in quantitative real-time RT-PCR, the level of *IL8 *gene expression after oxLDL treatment in M2 macrophages was markedly lower than those in M0 and M1 macrophages; and 7) *HMOX1 *gene expression levels were almost the same in all 3 subsets of macrophages even after oxLDL treatment.

The top genes expression of which was upregulated by oxLDL treatment in M2 but not M1 macrophages were highly correlated with the genes that were upregulated in M0 (Table 1). Moreover, the top altered ontology categories during oxLDL treatment in M2 macrophages were more similar to those of M0 than M1 (Figure 3). It has been reported that M-CSF-induced macrophages (M0 macrophages in the present study) have a similar transcriptional profile to M2 macrophages [10]. The transcriptional alteration during oxLDL treatment in M2 macrophages was also relatively similar to that in M0 macrophages but not M1 cells (Table 1 and Figure 3).

The data of hierarchical cluster analysis are shown in Figure 5. Commonly upregulated genes in all subsets of macrophages included some ARE-containing genes; *e.g*., *TRIM16*, *HMOX1*, *TXNRD1*, *GCLM*, and *DUSP1*. *TRIM16*, *HMOX1*, *TXNRD1*, *GCLM*, and *DUSP1 *encode tripartite motif-containing 16 (TRIM16), HO-1, thioredoxin reductase (Txnrd) 1, glutamate-cysteine ligase, modifier subunit (GCLM), and dual specificity phosphatase (DUSP) 1, respectively. These genes were upregulated during oxLDL treatment in all subsets of macrophages in the present study. ARE is a binding site for the transcription factor Nrf2 [20]. The Nrf2-ARE pathway plays a crucial role in protection against oxidative stress [21]. On exposure to oxidative stress, Nrf2 translocates to the nucleus, binds to the ARE [22], and activates the genes, including *TRIM16*, *HMOX1*, *TXNRD1*, *GCLM*, and *DUSP1*. These data were consistent with the recent report that oxidized phospholipids upregulated expression of ARE-containing genes in murine bone marrow-derived macrophages [19].

TGF-β has been suggested to have antiinflammatory properties [23], and it thought to be produced by alternatively activated macrophages [24]. Activation of M1 macrophages might be altered by M2-derived TGF-β. As TGF-β downregulates scavenger receptors, such as scavenger receptor type A (SR-A) I/II and CD36 [25], and upregulates ATP-binding cassette (ABC) transporters, ABCA1 and ABCG1 [26], TGF-β is also thought to have protective effects against the development of atherosclerosis. However, the contribution of TGF-β to the development of atherosclerosis is more complicated, taking account of clinical data. It is controversial whether TGF-β levels in blood from patients are positively or negatively correlated with cardiovascular disease [27]. In molecular network analysis, the molecules in the highest scoring network of M1 macrophages, but not M0 or M2, were related directly or indirectly to TGF-β1 (Figure 4). However, no molecules in the known TGF-β signal transduction pathway, including TGF-β receptors (TβRs) and SMADs, were altered by oxLDL treatment in this study. The results of cDNA microarray analysis (Figure 4) and real-time RT-PCR analysis (data not shown) indicated that oxLDL treatment slightly induced TGF-β1 gene expression in M1 macrophages. TGF-β generally plays an important role in maintaining normal vessel wall conditions, including the expression of contractile proteins in vascular smooth muscle cells (VSMCs) [28]. Under atherogenic conditions, however, TGF-β reduces extracellular matrix production from VSMCs and enhances leukocytes recruitment to atherosclerotic plaques, resulting in plaque rupture. Our results suggest that TGF-β-related molecules were affected by oxLDL stimulation, and that TGF-β promoted proinflammatory activities in M1 macrophages as in VSMCs. These findings suggest that oxLDL regulates the functions of M1 macrophages through an as yet unknown TGF-β-mediated cascade. It is therefore necessary to elucidate the detailed TGF-β-related functions regulated by oxLDL stimulation in various cells.

NF-κB is present in an inactive form bound to an inhibitor protein (I-κB) in the cytoplasm. On stimulation, NF-κB is released from I-κB, is translocated to the nucleus, and binds to the promoter DNA, followed by production of many types of inflammatory cytokine [29,30]. The NF-κB signaling pathway is known to be activated by oxLDL in a CD36-dependent manner [31]. Interestingly, cluster B included genes related to the NF-κB signaling pathway, such as NF-κB and PI3K (Figure 5 Table 3). The results of molecular network analysis indicated that oxLDL treatment induced upregulation of the growth factor receptor-mediated NF-κB signaling pathway in M1 but not M0 or M2 macrophages, while I-κB was upregulated in M0 and M2 but not M1 (Additional file 2). Thus, it seems that oxLDL stimulated the NF-κB signaling pathway specifically in M1.

There have been some reports partially conflicting with this study [10,11], probably due to differences in experimental conditions, such as oxLDL concentrations, TBARS levels, or duration of treatment. Further studies are required to determine whether M1 macrophages contribute to foam cell formation. In this study, we primarily measured mRNA levels, and all samples were obtained from healthy volunteers. Measurements of protein levels and data derived from atherosclerotic subjects should be included in the next study.

## Conclusions

The present study demonstrated the effects of oxLDL on transcriptional alterations in polarized macrophages. The data suggested that oxLDL uptake may affect TGF-β1- and NF-κB-mediated functions of M1 macrophages, but not M0 or M2 macrophages. It is likely that M1 macrophages characteristically respond to oxLDL. Further studies are required to evaluate the roles of TGF-β1- and NF-κB-mediated macrophage functions in the early stages of foam cell formation.

## Competing interests

The authors declare that they have no competing interests.

## Authors' contributions

KI, KS, and HD designed the study. KH participated at all stages and drafted the manuscript. CI conducted cDNA microarray analysis, HN performed flow cytometry analysis, and TK provided valuable help with cell cultures. KH, CI and HN analyzed the data together with KI, KS, and HD. KH, KI, and KS co-wrote the paper. All authors read and approved the final draft of the manuscript.

## Supplementary Material

Additional file 1 **All transcriptional profile**. After eliminated controls and backgrounds from all the probe sets on "3D-Gene" human oligo chip 25 k, the remained 24267 probe sets are listed. The values denote fold changes (log_2 _ratio) of normalized intensities during oxLDL treatment. Pink and blue cells indicate the genes showing a change in expression of >2-fold during oxLDL treatment as significantly up- or downregulated (log_2 _ratios were greater than +1 or less than -1). Genes intensities of which were less than 2^-4 ^are shown as blank cells. Click here for file

Additional file 2 **Molecular network focusing on the NF-κB signaling pathway**. This network consisted of the known NF-κB signaling pathway. The intensity of the node color indicates the degree of upregulation (red) or downregulation (blue). Nodes are displayed using various shapes represent the functional class of the gene product. Lines are displayed with various labels that describe the nature of the relationship between the nodes: A for Activation, M for Biochemical Modification, P for Phosphorylation/Dephosphorylation, PP for Protein - Protein binding, PR for Protein - RNA binding, RB for Regulation of Binding, T for Transcription, and TR for Translocation. The growth factor receptor-mediated NF-κB signaling pathway was upregulated in M1, but not in M0 or M2 macrophages. Click here for file
