# Supplementary material for: Different responses to oxidized low-density lipoproteins in human polarized macrophages
Source: Lipids Health Dis. 2011 Jan 4;10:1. doi: 10.1186/1476-511X-10-1 (PMC3022593; doi:10.1186/1476-511X-10-1)
Supplement: Additional file 2 — Molecular network focusing on the NF-κB signaling pathway. This network consisted of the known NF-κB signaling pathway. The intensity of the node color indicates the degree of upregulation (red) or downregulation (blue). Nodes are displayed using various shapes represent the functional class of the gene product. Lines are displayed with various labels that describe the nature of the relationship between the nodes: A for Activation, M for Biochemical Modification, P for Phosphorylation/Dephosphorylation, PP for Protein - Protein binding, PR for Protein - RNA binding, RB for Regulation of Binding, T for Transcription, and TR for Translocation. The growth factor receptor-mediated NF-κB signaling pathway was upregulated in M1, but not in M0 or M2 macrophages. [file 1476-511X-10-1-S2.PDF]

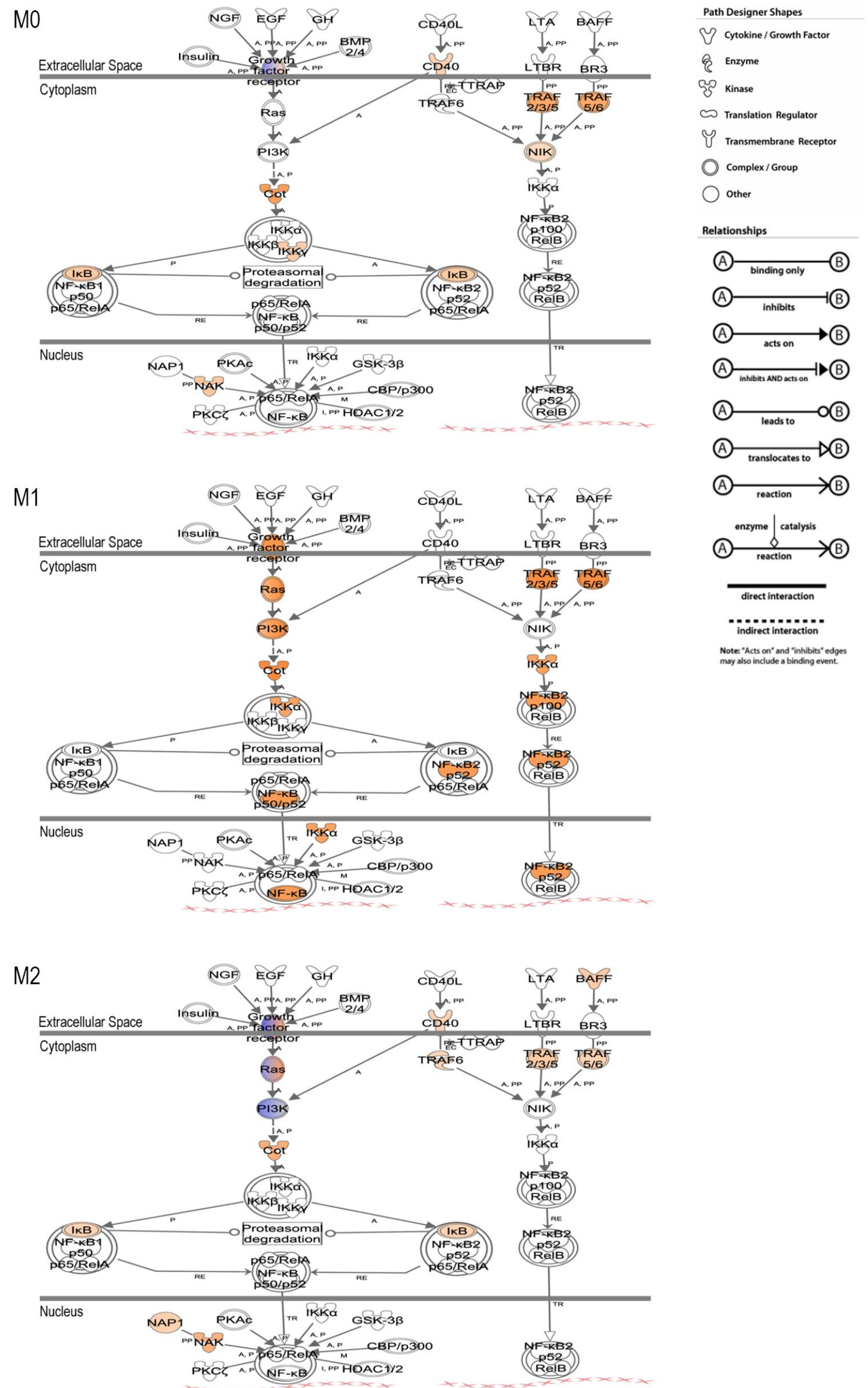
M0
M1
M2
Extracellular Space
Cytoplasm
Nucleus
NGF
EGF
GH
BMP 2/4
Insulin
Growth factor receptor
CD40L
CD40
TTRAP
TRAF6
LTA
LTBR
BAFF
BR3
TRAF 2/3/5
TRAF 5/6
Ras
PI3K
NIK
Cot
IKKα
IKKβ
IKKγ
NF-κB2 p100 RelB
NF-κB2 p52 RelB
IκB
NF-κB1 p50 p65/RelA
NF-κB2 p52 p65/RelA
Proteasomal degradation
p65/RelA NF-κB p50/p52
NAP1
PKAc
GSK-3β
NAK
CBP/p300
p65/RelA NF-κB
PKCζ
HDAC1/2
A, PP
A, P
A
P
PP
RE
TR
M
I, PP
Path Designer Shapes
Cytokine / Growth Factor
Enzyme
Kinase
Translation Regulator
Transmembrane Receptor
Complex / Group
Other
Relationships
binding only
inhibits
acts on
inhibits AND acts on
leads to
translocates to
reaction
enzyme
catalysis
direct interaction
indirect interaction
Note: "Acts on" and "inhibits" edges may also include a binding event.
